# Generalized immune activation as a direct result of activated CD4^+ ^T cell killing

**DOI:** 10.1186/jbiol194

**Published:** 2009-11-27

**Authors:** Rute Marques, Adam Williams, Urszula Eksmond, Andy Wullaert, Nigel Killeen, Manolis Pasparakis, Dimitris Kioussis, George Kassiotis

**Affiliations:** 1 Division of Immunoregulation, MRC National Institute for Medical Research, The Ridgeway, London NW7 1AA, UK; 2 Division of Molecular Immunology, MRC National Institute for Medical Research, The Ridgeway, London NW7 1AA, UK; 3 Institute for Genetics, University of Cologne, Zülpicher Strasse 47, 50674 Cologne, Germany; 4 Department of Microbiology and Immunology, University of California, San Francisco, CA 94143, USA; 5 Department of Immunobiology, Yale University School of Medicine, New Haven, CT 06520, USA

## Abstract

**Background:**

In addition to progressive CD4^+ ^T cell immune deficiency, HIV infection is characterized by generalized immune activation, thought to arise from increased microbial exposure resulting from diminishing immunity.

**Results:**

Here we report that, in a virus-free mouse model, conditional ablation of activated CD4^+ ^T cells, the targets of immunodeficiency viruses, accelerates their turnover and produces CD4^+ ^T cell immune deficiency. More importantly, activated CD4^+ ^T cell killing also results in generalized immune activation, which is attributable to regulatory CD4^+ ^T cell insufficiency and preventable by regulatory CD4^+ ^T cell reconstitution. Immune activation in this model develops independently of microbial exposure. Furthermore, microbial translocation in mice with conditional disruption of intestinal epithelial integrity affects myeloid but not T cell homeostasis.

**Conclusions:**

Although neither ablation of activated CD4^+ ^T cells nor disruption of intestinal epithelial integrity in mice fully reproduces every aspect of HIV-associated immune dysfunction in humans, ablation of activated CD4^+ ^T cells, but not disruption of intestinal epithelial integrity, approximates the two key immune alterations in HIV infection: CD4^+ ^T cell immune deficiency and generalized immune activation. We therefore propose activated CD4^+ ^T cell killing as a common etiology for both immune deficiency and activation in HIV infection.

See minireview http://www.jbiol.com/content/8/10/91

## Background

T lymphocyte numbers in the human body are kept constant by homeostatic mechanisms balancing cell gain and loss. These mechanisms eventually fail in HIV infection, which is characterized by progressive immune deficiency, because of loss of CD4^+ ^T cell function [1]. HIV infection is also associated with increased T cell turnover and activation, which extends to uninfected cells, resulting in a state of chronic generalized immune activation [2-5]. Indeed, the level of activation and turnover in CD8^+ ^T cells, which are not infected by HIV, can be higher than in CD4^+ ^T cells, and this is a powerful predictor of disease progression [2,4,5]. Early views of generalized immune activation as a compensatory mechanism to achieve T cell homeostasis after virus-mediated CD4^+ ^T cell destruction [6-8] have been replaced by alternative models in which immune activation is the cause, rather than the consequence, of CD4^+ ^T cell loss. In the latter models, immune activation is considered to be directly responsible for increased proliferation and death of both CD4^+ ^and CD8^+ ^T cells [9-11]. There is a strong positive correlation between T cell immune activation and CD4^+ ^T cell loss in HIV infection [12]. However, as the precise origin of generalized immune activation is still not fully understood, the direction of causality between CD4^+ ^T cell loss and immune activation remains unclear.

Immunodeficiency viruses are highly selective for activated/memory CD4^+ ^T cells owing to the restricted expression solely in these cells of CCR5, the co-receptor for HIV and simian immunodeficiency virus (SIV) [13,14], or CD134 (also called OX40 or Tumor necrosis factor receptor superfamily 4, TNFRSF4), the cellular receptor for feline immunodeficiency virus (FIV) [15]. This fraction of CD4^+ ^T cells is characterized by substantial heterogeneity and consists of T cells with distinct homeostatic behavior and functional role. The two major and best characterized subsets are antigen-experienced memory CD4^+ ^T cells and regulatory T (Treg) cells. Similarly to naïve CD4^+ ^T cells, Treg cells, which are equipped with immune-suppressive capacity, are generated in the thymus [16,17]. Newly generated Treg cells have a pre-activated phenotype and a considerable fraction also show higher turnover rates than naïve CD4^+ ^T cells in the periphery [18,19]. Peripheral Treg cell numbers are also regulated homeostatically. However, the requirements for peripheral maintenance of the Treg cell pool may differ from those for other CD4^+ ^T cell subsets, and precise knowledge of the relative contribution of thymic or peripheral generation to maintenance of Treg cell numbers remains incomplete [16,17]. Memory CD4^+ ^T cells are generated following the response of naïve CD4^+ ^T cells to infection or immunization in the periphery and mediate immunity to re-infection. However, in contrast to the naïve CD4^+ ^T cell pool, maintenance of which relies to a large extent on continuous thymic production, the memory CD4^+ ^T cell pool has considerable self-renewal capacity, regulated independently from the naïve CD4^+ ^T cell compartment, and can be maintained long-term in the absence of thymic function [20,21]. Although at the population level memory CD4^+ ^T cells are much longer lived than naïve CD4^+ ^T cells, at the individual-cell level memory CD4^+ ^T cells show a considerably higher turnover rate than relatively quiescent naïve CD4^+ ^T cells [20,22]. The high turnover rate within the memory CD4^+ ^T cell pool is thought to be driven, to a variable degree, by antigen and homeostatic cytokines [20].

Although memory CD4^+ ^T cells are the most frequent targets for HIV replication, they do not necessarily suffer the biggest loss during the chronic phase of infection. Indeed, the proportion of activated CCR5^+^CD4^+ ^T cells during HIV or SIV infection correlates strongly with the degree of pathogenesis. In contrast to their loss during progressive HIV-1 infection, CCR5^+^CD4^+ ^T cells are preserved in individuals who spontaneously control HIV-1 infection [23] and are even increased during the less pathogenic HIV-2 infection [24]. Similarly, CCR5^+^CD4^+ ^T cells are quickly lost during rapidly progressing SIV infection of Indian-origin rhesus macaques, but are increased in frequency during SIV infection of Chinese-origin macaques, characterized by much slower progression to disease [25]. The paradoxical increase in the proportion of CCR5^+^CD4^+ ^T cells during less pathogenic HIV and SIV infection is thought to result from robust replenishment of lost CD4^+ ^T cells as part of the physiological homeostatic process, and it may also be partly fueled by immune activation [11].

We have applied a reductionist approach to study the effect of depletion of activated CD4^+ ^T cells, the targets of immunodeficiency viruses, in a virus-free mouse model. We show here that conditional ablation of activated CD4^+ ^T cells greatly accelerates their turnover, with minimal apparent effect on their numbers, and results in CD4^+ ^T cell immune deficiency. More importantly, activated CD4^+ ^T cell killing in this model also results in generalized immune activation, independently of viral infection, reactivity to apoptotic T cells and microbial exposure. In contrast, we further show that generalized immune activation following activated CD4^+ ^T cell killing is due to an insufficiency of Treg cells.

## Results

### Conditional deletion of activated CD4^+ ^T cells

**Figure 1:**
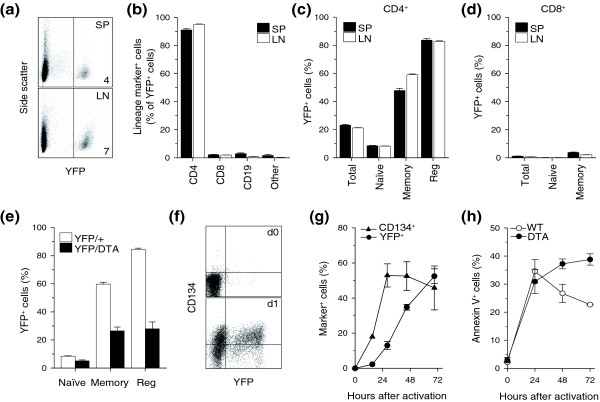
Specific targeting of memory and regulatory CD4^+ ^T cells by *Tnfrsf4*-driven Cre expression. **(a) **Activated YFP expression in a subset of splenic (SP) and lymph node cells (LN) isolated from *Tnfrsf4*^*Cre*/+ ^*R26*^*Yfp*/+ ^mice. Numbers within dot plots denote the percentage of YFP^+ ^cells. **(b) **Percentage (mean ± SEM, *n *= 6-9) of CD4^+^, CD8^+ ^or CD19^+ ^cells or cells negative for all three markers (other) in gated YFP^+ ^cells from the spleen and lymph nodes of *Tnfrsf4*^*Cre*/+^*R26*^*Yfp*/+ ^mice. **(c, d) **Percentage (mean ± SEM, *n *= 6-9) of YFP^+ ^cells in (c) total, naïve (CD44^lo^CD25^-^), memory (CD44^hi^CD25^-^) and regulatory (reg; CD25^+^) CD4^+ ^T cells and in (d) total, naïve (CD44^lo^CD25^-^) and memory (CD44^hi^CD25^-^) CD8^+ ^T cells, both from the spleen and lymph nodes of *Tnfrsf4*^*Cre*/+^*R26*^*Yfp*/+ ^mice. **(e) **Percentage (mean ± SEM, *n *= 4-8) of YFP^+ ^cells in naïve, memory and regulatory CD4^+ ^T cells from *Tnfrsf4*^*Cre*/+ ^*R26*^*Yfp*/+ ^(YFP/+) and *Tnfrsf4*^*Cre*/+ ^*R26*^*Yfp*/*Dta *^(YFP/DTA) mice. **(f) **Flow cytometric example of YFP and CD134 induction 1 day (d1) after *in vitro *stimulation of sorted naïve YFP^- ^CD4^+ ^T cells (d0) from *Tnfrsf4*^*Cre*/+ ^*R26*^*Yfp*/+ ^mice. **(g) **Percentage of YFP^+ ^and CD134^+ ^cells (mean ± SEM, *n *= 4-6) in CD4^+ ^T cells stimulated as in (f). **(h) **Percentage of annexin V^+ ^cells following *in vitro *activation of sorted naïve CD4^+ ^T cells from *Tnfrsf4*^*Cre*/+^*R26*^*Dta*/+ ^(DTA) or control *Tnfrsf4*^*Cre*/+^*R26*^+/+ ^(WT) mice.

To examine the consequences of activated CD4^+ ^T cell deletion for immune homeostasis, we generated a genetic mouse model in which activated CD4^+ ^T cells were killed in the absence of retroviral infection. Activated CD4^+ ^T cells were targeted by conditional gene activation mediated by *Tnfrsf4*-driven Cre recombinase expression [26]. Specificity for CD4^+ ^T cells was confirmed by activation of a yellow fluorescent protein (YFP) reporter gene in the *Gt(ROSA)26Sor *(*R26*) locus [27], which revealed that about 95% of YFP^+ ^cells were CD4^+ ^T cells (Figure 1a, b). YFP expression in *Tnfrsf4*^*Cre*/+ ^*R26*^*Yfp*/+ ^mice marked 55% and 80% of memory and regulatory CD4^+ ^T cells, respectively (Figure 1c). In contrast, the vast majority of naïve CD4^+ ^T cells (92%) and naïve and memory CD8^+ ^T cells (99% and 97%, respectively) were YFP^-^(Figure 1c, d). Conditional deletion of CD134-expressing CD4^+ ^T cells was achieved by Cre-mediated activation of a gene encoding diphtheria toxin fragment A (DTA) independently targeted into the *R26 *locus (Additional data file 1).

The efficiency of DTA-mediated T cell deletion was assessed in *Tnfrsf4*^*Cre*/+ ^*R26*^*Yfp*/*Dta *^heterozygous mice. In comparison with *Tnfrsf4*^*Cre*/+ ^*R26*^*Yfp*/+ ^mice, the proportion of YFP^+ ^memory and regulatory CD4^+ ^T cells in *Tnfrsf4*^*Cre*/+ ^*R26*^*Yfp*/*Dta *^mice was reduced by more than half (Figure 1e), suggesting that more than 50% of the cells that were tagged with YFP in the absence of DTA expression were killed on DTA activation. However, this analysis ignored the dynamic nature of T cell death and replacement. The relative presence of activated CD4^+ ^T cells and proportion of YFP^+ ^T cells in *Tnfrsf4*^*Cre*/+^*R26*^*Yfp*/*Dta *^mice reflected equilibrium between DTA-mediated killing, which would reduce, and homeostatic replacement, which would increase, the number of YFP^+ ^activated CD4^+ ^T cells, in addition to the relative kinetics of YFP and DTA induction following T cell activation. *In vitro *activated purified CD134^-^YFP^- ^naïve CD4^+ ^T cells from *Tnfrsf4*^*Cre*/+ ^*R26*^*Yfp*/+ ^mice began to express YFP by the first day of culture, with a delay of about 1 day relative to CD134 induction (Figure 1f, g). However, the effect of DTA activation on survival of *in vitro *activated naïve CD4^+ ^T cells from *Tnfrsf4*^*Cre*/+ ^*R26*^*Dta*/+ ^mice was not evident until the second day of culture (Figure 1h).

### Consequences of activated CD4^+ ^T cell killing for CD4^+ ^T cell homeostasis

**Figure 2:**
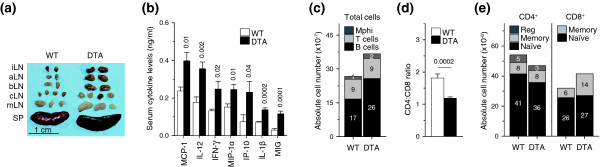
Immunological consequences of DTA-mediated deletion of CD134^+^CD4^+ ^T cells. **(a) **Size of inguinal (iLN), axillary (aLN), brachial (bLN), cervical (cLN), mesenteric (mLN) lymph nodes and spleen (SP) from *Tnfrsf4*^*Cre*/+ ^*R26*^*Dta*/+ ^(DTA) and littermate control *Tnfrsf4*^*Cre*/+ ^*R26*^+/+ ^(WT) mice. **(b) **Serum levels (mean ± SEM, *n *= 5-7) of MCP-1, IL-12 (p40), IFN-γ, MIP-1α, IP-10 (CXCL10), IL-1β and MIG (CXCL9) in the same mice. **(c) **Total numbers of B cells, T cells and macrophages (Mphi). *P *= 0.02 and *P *= 0.03 for total cells and B cells, respectively. **(d) **CD4:CD8 ratio. **(e) **Total numbers (mean, *n *= 9-12) of naïve, memory and regulatory (reg) CD4^+ ^T cells and naïve and memory CD8^+ ^T cells. *P *= 0.0008 for regulatory CD4^+ ^T cells; *P *= 0.04 for total CD8^+ ^T cells; *P *= 0.0003 for memory CD8^+ ^T cells. Numbers within bars in (c, e) denote the absolute number, ×10^-7 ^and ×10^-6^, respectively, of each cell type.

To determine whether activated CD4^+ ^T cell deletion had any impact on lymphocyte population dynamics, we analyzed lymphoid organ cellularity and composition. We observed significant systemic lymph node enlargement in *Tnfrsf4*^*Cre*/+ ^*R26*^*Dta*/+ ^mice compared with control *Tnfrsf4*^*Cre*/+ ^*R26*^+/+ ^mice (Figure 2a), which was also associated with elevated serum levels of several proinflammatory mediators (Figure 2b). Spleen size was not appreciably affected (Figure 2a). We thus calculated the total size of lymphocyte and myeloid populations as the sum of the cellular contents of the spleen and of inguinal, axillary, brachial, mesenteric and superficial cervical lymph nodes. B cells, but not T cells or myeloid cells, were significantly more numerous in *Tnfrsf4*^*Cre*/+ ^*R26*^*Dta*/+ ^mice than in control *Tnfrsf4*^*Cre*/+ ^*R26*^+/+ ^mice (Figure 2c).

Deletion of activated CD4^+ ^T cells resulted in a substantial systemic drop in the CD4:CD8 ratio (Figure 2d). Remarkably, compared with control mice, total CD4^+ ^T cell numbers in *Tnfrsf4*^*Cre*/+^*R26*^*Dta*/+ ^mice were only marginally reduced and remained stable throughout a 6-month observation period (Figure 2e). To assess whether activated CD4^+ ^T cell numbers were selectively reduced in *Tnfrsf4*^*Cre*/+ ^*R26*^*Dta*/+ ^mice, we determined the composition of the CD4^+ ^T cell pool. Numbers of naïve CD4^+ ^T cells and, notably, of memory CD4^+ ^T cells were similar between *Tnfrsf4*^*Cre*/+ ^*R26*^*Dta*/+ ^and control *Tnfrsf4*^*Cre*/+ ^*R26*^+/+ ^mice (Figure 2e), whereas numbers of regulatory CD4^+ ^T cells were reduced by about 40% in *Tnfrsf4*^*Cre*/+ ^*R26*^*Dta*/+ ^mice (Figure 2e). In contrast to CD4^+ ^T cells, total numbers of CD8^+ ^T cells were elevated in *Tnfrsf4*^*Cre*/+ ^*R26*^*Dta*/+ ^mice compared with *Tnfrsf4*^*Cre*/+ ^*R26*^+/+ ^mice, resulting from a systemic expansion exclusively of memory CD8^+ ^T cells (Figure 2e), which was primarily responsible for the systemic reduction in the CD4:CD8 ratio.

**Figure 3:**
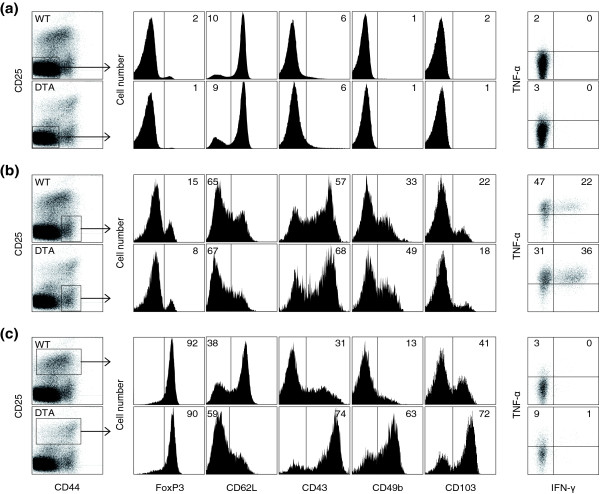
Effect of CD134^+^CD4^+ ^T cell killing on the phonotype of CD4^+ ^T cells. **(a) **Naïve, **(b) **memory and **(c) **regulatory CD4^+ ^T cell expression of FoxP3 and activation markers, and production of cytokines following *in vitro *re-stimulation. Numbers within the plots represent the percentage of CD4^+ ^T cells that were positive for each marker. Plots are representative of 4-7 mice per group.

Preservation of CD4^+ ^T cell numbers despite killing of activated CD4^+ ^T cells in *Tnfrsf4*^*Cre*/+ ^*R26*^*Dta*/+ ^mice suggested increased replenishment, which would be associated with functional and phenotypic activation. Phenotypic differences between naïve or memory CD4^+ ^T cells in *Tnfrsf4*^*Cre*/+ ^*R26*^*Dta*/+ ^and those in control *Tnfrsf4*^*Cre*/+ ^*R26*^+/+ ^mice were largely unremarkable, with modest increases in expression of cytokines and of the activation markers CD43 and CD49b in memory CD4^+ ^T cells isolated from *Tnfrsf4*^*Cre*/+ ^*R26*^*Dta*/+ ^mice (Figure 3a, b). In contrast to regulatory CD4^+ ^T cells from control mice, those from *Tnfrsf4*^*Cre*/+ ^*R26*^*Dta*/+ ^mice showed a highly activated phenotype, characterized by downregulation of CD62L and upregulation of CD44, CD43, CD49b and CD103 (Figure 3c). Thus, DTA-mediated destruction of activated CD4^+ ^T cells had a significant effect on regulatory CD4^+ ^T cell numbers and activation state, but little apparent effect on memory CD4^+ ^T cells.

**Figure 4:**
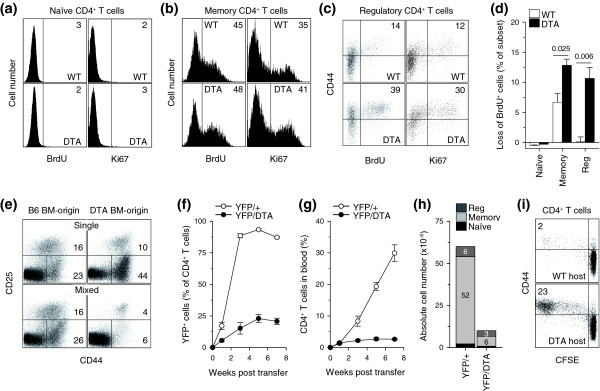
Effect of DTA-mediated deletion of memory and regulatory CD4^+ ^T cells on CD4^+ ^T cell homeostasis. **(a) **Naïve, **(b) **memory and **(c) **regulatory CD4^+ ^T cell BrdU incorporation during a 6-day administration period, and expression of Ki67 nuclear antigen. Numbers within the plots denote the percentage of positive CD4^+ ^T cells and are representative of 4-6 mice per group. *P *= 0.011 for Ki67 staining in memory CD4^+ ^T cells; *P *= 0.006 for BrdU incorporation and *P *= 0.0004 for Ki67 staining in regulatory CD4^+ ^T cells. **(d) **Loss of BrdU^+ ^naïve, memory or regulatory CD4^+ ^T cells 3 days after cessation of a 6-day BrdU administration period. Values are the percentage (± SEM) of BrdU^+ ^cells in each subset on day 3 after cessation of BrdU administration minus the percentage of BrdU^+ ^cells at the peak (day 6 of the administration period), and are representative of three mice per group. **(e) **CD45.2^+^*Tnfrsf4*^*Cre*/+ ^*R26*^*Dta*/+ ^(DTA) and CD45.1^+^C57BL/6 (B6) bone marrow (BM) cells were injected separately (single) or mixed together at 1:1 ratio (mixed) into non-irradiated *Rag1*^-/- ^recipients and lymphoid organs were analyzed 12 weeks later. CD25 and CD44 expression in gated DTA BM-origin or B6 BM-origin CD4^+ ^T cells in these recipients is shown. Numbers within the plots denote the percentage of positive CD4^+ ^T cells and are representative of 4-8 mice per group analyzed in two independent experiments. **(f-h) **1 × 10^6 ^purified CD4^+ ^T cells from *Tnfrsf4*^*Cre*/+^*R26*^*Yfp*/+ ^(YFP/+) or *Tnfrsf4*^*Cre*/+^*R26*^*Yfp*/*Dta *^(YFP/DTA) mice were adoptively transferred into *Rag1*^-/- ^recipients and followed over time. (f) Percentage of YFP^+ ^cells in CD4^+ ^T cells in the blood. (g) Percentage of CD4^+ ^T cells in blood mononuclear cells. (h) Total numbers of naïve, memory and regulatory (reg) CD4^+ ^T cells in lymphoid organs at the end of the 7-week observation period. *P *< 0.0001 for memory CD4^+ ^T cells; *P *= 0.006 for regulatory CD4^+ ^T cells. Values in (f-h) are the means (± SEM) of five mice per group. Numbers within bars in (h) denote the absolute number, ×10^-6^, of each cell type. **(i) **5 × 10^6 ^purified naïve (CD44^lo^CD25^-^) CD45.1^+ ^CFSE-labeled wild-type CD4^+ ^T cells were adoptively transferred into *Tnfrsf4*^*Cre*/+^*R26*^*Dta*/+ ^(DTA host) and control *Tnfrsf4*^*Cre*/+^*R26*^+/+ ^(WT host) recipient mice. CFSE dilution and CD44 expression on gated CD45.1^+ ^donor CD4^+ ^T cells isolated from the spleens and lymph nodes of recipients 6 days after transfer are shown. Numbers within the plots denote the percentage of CFSE^-^CD44^hi^CD4^+ ^T cells and are representative of three mice per group.

Memory CD4^+ ^T cells, under physiological conditions, display higher turnover rates, self-renewal potential and activation profile than either naïve or regulatory CD4^+ ^T cells [1]. Indeed, naïve CD4^+ ^T cells from either *Tnfrsf4*^*Cre*/+ ^*R26*^*Dta*/+ ^or control mice showed little evidence for cell division assessed either by incorporation of bromodeoxyuridine (BrdU) or staining with the Ki67 antibody (Figure 4a). In contrast, population turnover rates were very high in memory CD4^+ ^T cells from both *Tnfrsf4*^*Cre*/+ ^*R26*^*Dta*/+ ^and control mice (Figure 4b). Ki67 staining, but not BrdU incorporation, in memory CD4^+ ^T cells from *Tnfrsf4*^*Cre*/+^*R26*^*Dta*/+ ^mice was elevated in comparison with that in memory CD4^+ ^T cells from control mice (Figure 4b). Moreover, regulatory CD4^+ ^T cells had a significantly higher turnover rate in *Tnfrsf4*^*Cre*/+ ^*R26*^*Dta*/+ ^mice than in control mice, which approached the high turnover rate of memory CD4^+ ^T cells (Figure 4c).

Comparable representation of BrdU^+ ^memory CD4^+ ^T cells in both *Tnfrsf4*^*Cre*/+ ^*R26*^*Dta*/+ ^and control mice at the end of the 6-day BrdU pulsing period (Figure 4b) seemed discordant with the expected elevated turnover of memory CD4^+ ^T cells in *Tnfrsf4*^*Cre*/+ ^*R26*^*Dta*/+ ^mice. However, in memory CD4^+ ^T cells from *Tnfrsf4*^*Cre*/+ ^*R26*^*Dta*/+ ^mice, increased BrdU incorporation would be masked by increased DTA-mediated death of the proliferating cells during the pulsing period. We therefore examined the fate of BrdU^+ ^memory CD4^+ ^T cells 3 days after termination of BrdU administration (chase period). In contrast to the opposing action of cell proliferation and death, which would increase or decrease, respectively, the percentage of BrdU^+ ^cells during the pulsing period, cell proliferation, by dilution of BrdU label, and cell death would both decrease the percentage of BrdU^+ ^cells during the chase period. Indeed, almost twice as many BrdU^+ ^memory CD4^+ ^T cells were lost during the 3-day chase period in *Tnfrsf4*^*Cre*/+^*R26*^*Dta*/+ ^mice as in control mice (Figure 4d). Consistent with the Ki67 staining, the difference in the loss of BrdU^+ ^cells was even more pronounced in regulatory CD4^+ ^T cells (Figure 4d). These data together suggested that memory CD4^+ ^T cells, and to a higher degree regulatory CD4^+ ^T cells, had significantly elevated turnover rates in *Tnfrsf4*^*Cre*/+ ^*R26*^*Dta*/+ ^mice than in control mice.

To further reveal the full extent of memory CD4^+ ^T cell killing in *Tnfrsf4*^*Cre*/+ ^*R26*^*Dta*/+ ^mice, we generated mixed bone marrow chimeras. Compared with immunodeficient mice reconstituted with wild-type bone marrow alone, those reconstituted with *Tnfrsf4*^*Cre*/+ ^*R26*^*Dta*/+ ^bone marrow alone showed a paradoxical increase in memory CD44^+^CD4^+ ^T cells and a small reduction in regulatory CD25^+^CD4^+ ^T cells (Figure 4e). In contrast, mice reconstituted with a mixture of wild-type and *Tnfrsf4*^*Cre*/+ ^*R26*^*Dta*/+ ^bone marrow had a severe reduction in memory and regulatory CD4^+ ^T cell numbers of *Tnfrsf4*^*Cre*/+ ^*R26*^*Dta*/+ ^origin (Figure 4e). Comparison of the *Tnfrsf4*^*Cre*/+^*R26*^*Dta*/+^:wild-type ratio in thymocyte and peripheral lymphocyte subsets confirmed a significant selective loss of *Tnfrsf4*^*Cre*/+ ^*R26*^*Dta*/+^-origin memory and regulatory CD4^+ ^T cells (Additional data file 2). Thus, although both subsets were being killed with equal efficiency by DTA activation in *Tnfrsf4*^*Cre*/+ ^*R26*^*Dta*/+ ^mice, regulatory CD4^+ ^T cells were incompletely replenished, whereas the loss of memory CD4^+ ^T cells was overcompensated.

Continuous replenishment of memory CD4^+ ^T cells in *Tnfrsf4*^*Cre*/+ ^*R26*^*Dta*/+ ^mice could be due to constant recruitment of new cells from either memory CD4^+ ^T cells that did not express CD134 or naïve CD4^+ ^T cells, thymic production and peripheral numbers of which were minimally affected in these mice. To examine the contribution of thymic T cell production and of naïve CD4^+ ^T cells to the preservation of memory CD4^+ ^T cell numbers in *Tnfrsf4*^*Cre*/+ ^*R26*^*Dta*/+ ^mice, we infused purified CD4^+ ^T cells into lymphopenic recipients (adoptive transfer). *Rag1*^-/- ^mice, which are lymphopenic due to genetic deficiency in the V(D)J recombination activation gene *Rag1 *that precludes generation of lymphocytes, were chosen as recipients. Adoptive transfer of *Tnfrsf4*^*Cre*/+ ^*R26*^*Yfp*/+ ^CD4^+ ^T cells revealed that T cell proliferation in lymphopenic *Rag1*^-/- ^recipients was sufficient to drive full activation, as nearly all of the transferred CD4^+ ^T cells expressed the YFP reporter within 3 weeks of transfer (Figure 4f). In contrast, the proportion of YFP^+ ^cells in transferred *Tnfrsf4*^*Cre*/+ ^*R26*^*Yfp*/*Dta *^CD4^+ ^T cells that could activate both YFP and DTA remained low throughout the 7-week observation period (Figure 4f), similar to the low percentage of YFP^+ ^memory CD4^+ ^T cells found in donor *Tnfrsf4*^*Cre*/+^*R26*^*Yfp*/*Dta *^mice (Figure 1e). More importantly, in contrast to *Tnfrsf4*^*Cre*/+ ^*R26*^*Yfp*/+ ^CD4^+ ^T cells, which progressively expanded over time in *Rag1*^-/- ^recipients (Figure 4g), numbers of transferred *Tnfrsf4*^*Cre*/+^*R26*^*Yfp*/*Dta *^CD4^+ ^T cells remained low in the blood throughout the observation period (Figure 4g) and in the lymphoid organs of *Rag1*^-/- ^recipients 7 weeks after transfer (Figure 4h). Failure of *Tnfrsf4*^*Cre*/+ ^*R26*^*Yfp*/*Dta *^memory CD4^+ ^T cells to accumulate in the setting of naïve CD4^+ ^T cell deficiency demonstrated the requirement for recruitment of naïve CD4^+ ^T cells in order to maintain the pool of memory CD4^+ ^T cells.

To directly visualize the recruitment of naïve CD4^+ ^T cells into the memory pool of *Tnfrsf4*^*Cre*/+ ^*R26*^*Dta*/+ ^mice, we adoptively transferred purified carboxyfluorescein succinimidyl ester (CFSE)-labeled naïve (CD44^lo^CD25^-^) CD4^+ ^T cells from wild-type donor mice expressing the allotypic marker CD45.1. As expected, naïve CD4^+ ^T cells failed to proliferate or activate within 6 days of transfer into control *Tnfrsf4*^*Cre*/+ ^*R26*^+/+ ^mice (Figure 4i). In contrast, a substantial proportion of the progeny of transferred naïve CD4^+ ^T cells had divided extensively and acquired CD44 expression during the same time in *Tnfrsf4*^*Cre*/+^*R26*^*Dta*/+ ^mice (Figure 4i). Together, these observations support a model in which both memory and regulatory CD4^+ ^T cells are being killed with equal efficiency by expression of DTA in *Tnfrsf4*^*Cre*/+ ^*R26*^*Dta*/+ ^mice. However, as long as production and maintenance of naïve CD4^+ ^T cells is unaffected, the continual loss of memory but not regulatory CD4^+ ^T cells can be efficiently compensated for by continual recruitment of naïve CD4^+ ^T cells.

### Effect of activated CD4^+ ^T cell killing on immune competence

**Figure 5:**
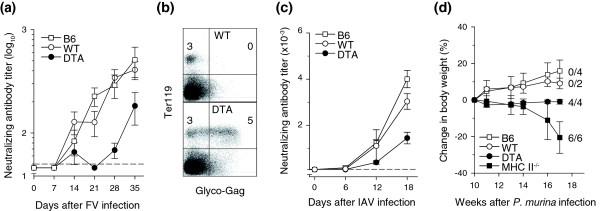
Effect of activated CD4^+ ^T cell killing on immune competence. **(a) **Serum titers (mean ± SEM, *n *= 5-7) of neutralizing antibodies (nAb) in FV-infected DTA, wild-type (WT) and B6 mice. *P *≤ 0.018 and *P *≤ 0.007 on days 21 and 28, respectively, between DTA mice and either WT or B6 mice. **(b) **FV-encoded glyco-Gag on the surface of infected erythroid precursor (Ter119^+^) cells in the same mice. Glyco-Gag^+ ^Ter119^+ ^cells were detected in four out of seven DTA mice. **(c) **Serum titers (mean ± SEM, *n *= 6-9) of nAb in IAV-infected DTA, WT and B6 mice. *P *= 0.002 between DTA and WT mice and *P *= 0.00003 between DTA and B6 mice on day 18. **(d) **Body weight changes in *Pneumocystis murina*-infected DTA, WT, B6 and MHC II-deficient (MHC II^-/-^) mice. *P *≤ 0.04 between DTA and WT or B6 mice on weeks 16 and 17 after infection for body weight changes. Numbers within the plot denote the ratio of mice tested positive for *P. murina *at the end of the observation period (*P *= 0.004 between DTA and control mice).

To investigate whether the accelerated death and replenishment of memory CD4^+ ^T cells compromised immune competence, we evaluated CD4^+ ^T cell function in *Tnfrsf4*^*Cre*/+ ^*R26*^*Dta*/+ ^mice. In comparison with *Tnfrsf4*^*Cre*/+ ^*R26*^+/+ ^littermates and C57BL/6 (B6) control mice, which showed a strong neutralizing antibody (nAb) response to and effectively contained infection with Friend virus (FV), a retrovirus that causes persistent infection in mice, the FV-specific nAb response was undetectable in four out of seven *Tnfrsf4*^*Cre*/+^*R26*^*Dta*/+ ^mice and significantly delayed in the rest (Figure 5a). Defective FV-specific nAb response also correlated with inability of *Tnfrsf4*^*Cre*/+ ^*R26*^*Dta*/+ ^mice to control FV replication (Figure 5b). Furthermore, following acute influenza A virus (IAV) infection, titers of CD4^+ ^T cell-dependent virus-neutralizing antibodies (nAbs) in the serum of *Tnfrsf4*^*Cre*/+^*R26*^*Dta*/+ ^mice were reduced to 48% and to 36% of those in *Tnfrsf4*^*Cre*/+ ^*R26*^+/+ ^littermates and B6 mice, respectively (Figure 5c). Titration of CD4^+ ^T cells into T cell-deficient mice revealed that this degree of reduction in IAV-specific nAb titers corresponded to about 80% loss of CD4^+ ^T cells (Additional data file 3). Lastly, in contrast to *Tnfrsf4*^*Cre*/+^*R26*^+/+ ^and B6 mice, which effectively cleared experimental infection with *Pneumocystis murina*, *Tnfrsf4*^*Cre*/+^*R26*^*Dta*/+ ^mice remained persistently infected and failed to thrive, with susceptibility intermediate between T cell-replete and MHC II-deficient mice, which lacked CD4^+ ^T cells (Figure 5d). These results showed that, despite the presence of normal numbers of CD4^+ ^T cells, *Tnfrsf4*^*Cre*/+ ^*R26*^*Dta*/+ ^mice were CD4^+ ^T cell immune deficient.

### Consequences of activated CD4^+ ^T cell killing for CD8^+ ^T cell homeostasis

**Figure 6:**
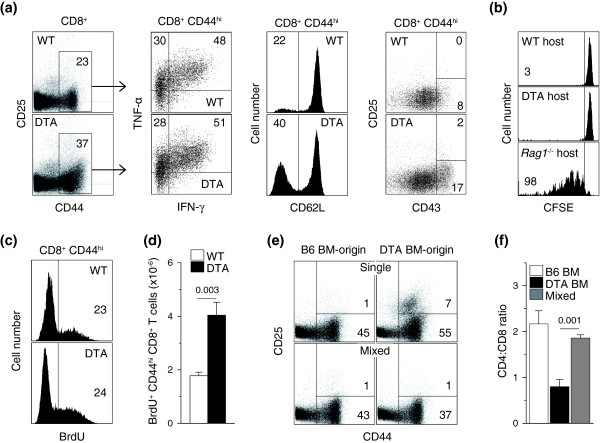
Effect of DTA-mediated deletion of CD134^+^CD4^+ ^T cells on CD8^+ ^T cell homeostasis. **(a) **TNF-α and IFN-γ production and CD62L, CD43 and CD25 expression in gated memory CD44^hi^CD8^+ ^T cells from *Tnfrsf4*^*Cre*/+^*R26*^*Dta*/+ ^(DTA) and littermate control *Tnfrsf4*^*Cre*/+ ^*R26*^+/+ ^(WT) mice (*n *= 5-12). Cytokine production was assessed following 4-h *in vitro *stimulation of total spleen and lymph node cells. **(b) **CFSE dilution profiles of purified naïve (CD44^lo^) CD45.1^+ ^CFSE-labeled wild-type CD8^+ ^T cells 6 days following adoptive transfer into *Tnfrsf4*^*Cre*/+ ^*R26*^*Dta*/+ ^(DTA host), control *Tnfrsf4*^*Cre*/+ ^*R26*^+/+ ^(WT host) or lymphopenic *Rag1*^-/- ^recipient mice (*Rag1*^-/- ^host). Numbers within the plot represent the mean percentage of CFSE^- ^donor CD8^+ ^T cells in three mice per group. **(c) **Percentage of BrdU^+ ^cells in memory CD44^hi^CD8^+ ^T cells and **(d) **absolute number (mean ± SEM, *n *= 4) of BrdU^+ ^cells in total CD8^+ ^T cells following a 6-day period of BrdU administration. **(e) **CD44 and CD25 expression in gated CD8^+ ^T cells of either DTA BM origin or B6 BM origin and **(f) **CD4:CD8 ratio (± SEM) in *Rag1*^-/- ^recipients reconstituted with either DTA or CD45.1^+ ^B6 bone marrow (single) or a 1:1 mixture of DTA and CD45.1^+ ^B6 bone marrow (mixed) (*n *= 4-7).

CD8^+ ^T cells in HIV infection are increased in numbers and also have a higher turnover rate and activation state [2,4,5]. This could result from either a composition change from quiescent naïve to activated memory CD8^+ ^T cells and/or further activation within the memory pool. Following *in vitro *stimulation of total spleen and lymph node cells, the expanded population of memory CD8^+ ^T cells in *Tnfrsf4*^*Cre*/+^*R26*^*Dta*/+ ^mice showed a pattern of cytokine production similar to that in control mice (Figure 6a), indicating that they were functionally intact. Compared with the relatively homogeneous memory CD8^+ ^T cell pool in control mice, memory CD8^+ ^T cells in *Tnfrsf4*^*Cre*/+ ^*R26*^*Dta*/+ ^mice contained higher numbers of recently activated/effector CD8^+ ^T cells, characterized by downregulation of CD62L and upregulation of CD43 expression (Figure 6a). To examine whether or not the expansion of the memory CD8^+ ^T cell pool in *Tnfrsf4*^*Cre*/+ ^*R26*^*Dta*/+ ^mice was due to recruitment of a greater fraction of naïve CD8^+ ^T cells, we adoptively purified CD45.1^+ ^naïve (CD44^lo^) CD8^+ ^T cells labeled with CFSE from wild-type donor mice. Naïve CD8^+ ^T cells remained undivided, as evident from the retention of CFSE label 6 days after transfer into either *Tnfrsf4*^*Cre*/+ ^*R26*^*Dta*/+ ^or control mice (Figure 6b). In contrast, naïve CD8^+ ^T cells divided extensively in lymphopenic *Rag1*^-/- ^recipients (Figure 6b). This finding suggested that naïve CD8^+ ^T cells were not constantly recruited into the memory pool of *Tnfrsf4*^*Cre*/+ ^*R26*^*Dta*/+ ^mice. The proportion of proliferating cells in memory CD8^+ ^T cells, assessed by BrdU incorporation during a 6-day administration period, was similarly high in *Tnfrsf4*^*Cre*/+ ^*R26*^*Dta*/+ ^and control mice (Figure 6c). However, because of the difference in memory CD8^+ ^T cell pool size, absolute numbers of BrdU^+ ^memory CD8^+ ^T cells were significantly higher in *Tnfrsf4*^*Cre*/+ ^*R26*^*Dta*/+ ^mice than in control mice (Figure 6d). Thus, the expanded memory CD8^+ ^T cell population in *Tnfrsf4*^*Cre*/+ ^*R26*^*Dta*/+ ^mice seemed to be maintained by elevated turnover within the memory pool.

We next confirmed that expansion and activation of memory CD8^+ ^T cells in *Tnfrsf4*^*Cre*/+ ^*R26*^*Dta*/+ ^mice was a consequence of accelerated turnover of activated CD4^+ ^T cells. In mice reconstituted with a mixture of wild-type and *Tnfrsf4*^*Cre*/+^*R26*^*Dta*/+ ^bone marrow, in which CD4^+ ^T cell homeostasis is restored by wild-type CD4^+ ^T cells (Figure 4e), memory CD8^+ ^T cells of either wild-type or *Tnfrsf4*^*Cre*/+ ^*R26*^*Dta*/+ ^origin were comparable, with no signs of activation (Figure 6e) or competitive disadvantage (Additional data file 2), and CD4:CD8 ratios were restored (Figure 6f). Furthermore, memory CD8^+ ^T cells in MHC II-deficient mice contained higher percentages of CD62L^- ^and CD43^+ ^activated/effector CD8^+ ^T cells than wild-type mice, despite proportional increases in numbers of both naïve and memory CD8^+ ^T cells (Additional data file 4). Thus, memory CD8^+ ^T cell activation and expansion in *Tnfrsf4*^*Cre*/+ ^*R26*^*Dta*/+ ^mice resulted from CD4^+ ^T cell insufficiency, rather than a cell-autonomous effect.

### Causes of immune activation following activated CD4^+ ^T cell depletion

Accelerated turnover of activated CD4^+ ^T cells could trigger generalized immune activation by several distinct mechanisms, including self-reactivity to apoptotic CD4^+ ^T cells [28], translocation of microbial products into the intestinal mucosa as a result of local effector CD4^+ ^T cell depletion, and loss of regulatory CD4^+ ^T cell activity [29,30]. Reactivity to apoptosis-related self peptides could be excluded as the cause of CD8^+ ^T cell activation in *Tnfrsf4*^*Cre*/+^*R26*^*Dta*/+ ^mice because activation of these cells was not observed in mixed bone marrow chimeras in the presence of wild-type CD4^+ ^T cells (Figure 6e), despite continuous apoptosis of *Tnfrsf4*^*Cre*/+ ^*R26*^*Dta*/+ ^CD4^+ ^T cells.

**Figure 7:**
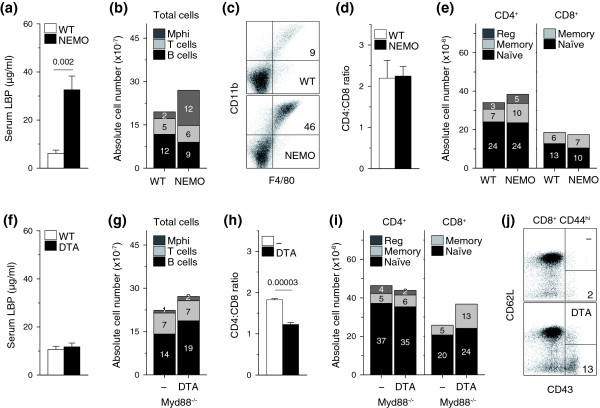
Influence of microbial exposure on immune homeostasis. **(a) **Serum LBP levels (mean ± SEM, *n *= 6-9) in *Ikbkg*^*fl*/*Y*^*Vil-Cre *(NEMO) and littermate control *Ikbkg*^*fl*/*Y*^(WT) mice. The number above the bars is the *P*-value. **(b) **Total numbers (mean, *n *= 8-9) of B cells, T cells and macrophages (Mphi) in *Ikbkg*^*fl*/*Y *^*Vil-Cre *(NEMO) and littermate control *Ikbkg*^*fl*/*Y *^(WT) mice. *P *= 0.0007 for macrophages. **(c) **Flow cytometric example of CD11b^+^F4/80^+ ^macrophage expansion in the spleen of NEMO mice, compared with WT mice. **(d) **CD4:CD8 ratio (± SEM) in the same mice. **(e) **Total numbers of naïve, memory and regulatory (reg) CD4^+ ^T cells and naïve and memory CD8^+ ^T cells from NEMO and control WT mice. **(f) **Serum LBP levels (mean ± SEM, *n *= 9-13) in *Tnfrsf4*^*Cre*/+ ^*R26*^*Dta*/+ ^(DTA) and control *Tnfrsf4*^*Cre*/+ ^*R26*^+/+ ^(WT) mice. **(g) **Total numbers (mean, *n *= 4-6) of B cells, T cells and macrophages (Mphi), **(h) **CD4:CD8 ratio and **(i) **total numbers of naïve, memory and regulatory (reg) CD4^+ ^T cells and naïve and memory CD8^+ ^T cells in *Tnfrsf4*^*Cre*/+ ^*R26*^*Dta*/+^*MyD88*^-/- ^(DTA) and littermate control *Tnfrsf4*^*Cre*/+ ^*R26*^+/+ ^*MyD88*^-/- ^(-) mice. *P *= 0.035 for total cells; *P *= 0.015 for B cells; *P *= 0.0003 for regulatory CD4^+ ^T cells; *P *= 0.018 for total CD8^+ ^T cells; and *P *= 0.0003 for memory CD8^+ ^T cells. **(j) **CD62L and CD43 expression in gated memory CD44^hi^CD8^+ ^T cells from the same mice. Numbers within bars denote the absolute number, ×10^-7 ^in (b, g), and ×10^-6 ^in (e, i), of each cell type.

Although we found no evidence for intestinal pathology in *Tnfrsf4*^*Cre*/+ ^*R26*^*Dta*/+ ^mice, it could be that a small degree of histologically undetectable bacterial translocation was occurring. To answer this question, we examined the effect of microbial translocation on the immune system of *Ikbkg*^*fl*/*Y*^*Vil-Cre *mice, in which intestinal epithelial cell-specific deletion of NFκB essential modulator (NEMO, encoded by *Ikbkg*) leads to epithelial cell apoptosis, translocation of bacteria into the mucosa and myeloid differentiation primary response gene 88 (MyD88)-dependent intestinal inflammation and colitis [31]. Consistent with disruption of the intestinal epithelial barrier, *Ikbkg*^*fl*/*Y*^*Vil-Cre *mice showed significantly elevated serum levels of lipopolysaccharide-binding protein (LBP), a surrogate marker for the systemic presence of bacterial lipopolysaccharide, in comparison with control mice (Figure 7a). Numbers of B cells, CD4^+ ^and CD8^+ ^T cell subsets and the CD4:CD8 ratio were comparable between *Ikbkg*^*fl*/*Y*^*Vil-Cre *mice and control *Ikbkg*^*fl*/*Y *^mice (Figure 7b-e) and sporadic lymphocyte expansions at inflamed mesenteric lymph nodes [31] were at the expense of splenic counterparts (Figure 7e; and data not shown). Moreover, T cell turnover was not systemically increased in *Ikbkg*^*fl*/*Y *^*Vil-Cre *mice compared with control mice, and an increase in BrdU incorporation in memory CD8^+ ^T cells in mesenteric lymph nodes was balanced by a proportional decrease in the spleen (Additional data file 5). In contrast to lymphocytes, numbers of cells expressing the myeloid markers CD11b^+ ^and F4/80^+ ^were dramatically elevated in all secondary lymphoid organs of *Ikbkg*^*fl*/*Y*^*Vil-Cre *mice and outnumbered B cells or T cells (Figure 7b-e). Thus, microbial translocation in this mouse model was associated with systemic expansion of myeloid cells, but was not sufficient for T cell activation.

We next examined the contribution of microbial exposure to the immune activation status of *Tnfrsf4*^*Cre*/+ ^*R26*^*Dta*/+ ^mice. In agreement with the absence of obvious intestinal pathology, serum levels of LBP in *Tnfrsf4*^*Cre*/+ ^*R26*^*Dta*/+ ^mice were found to be similar to those in control mice (Figure 7f), arguing against a requirement for microbial translocation for immune activation in *Tnfrsf4*^*Cre*/+ ^*R26*^*Dta*/+ ^mice. To formally exclude any potential contribution of microbial exposure, we rendered *Tnfrsf4*^*Cre*/+ ^*R26*^*Dta*/+ ^mice deficient in MyD88. In contrast to the essential role of MyD88 in microbial recognition driving disease in *Ikbkg*^*fl*/*Y *^*Vil-Cre *mice [31], MyD88 deficiency had no effect on immune activation in *Tnfrsf4*^*Cre*/+^*R26*^*Dta*/+ ^mice, which still experienced lymph node enlargement with accumulation of B cells, expansion of memory CD8^+ ^T cells, particularly of the activated CD62L^-^CD43^+ ^phenotype, and a drop in the CD4:CD8 ratio (Figure 7g-j). Thus, MyD88-dependent microbial exposure was not necessary for immune activation in *Tnfrsf4*^*Cre*/+^*R26*^*Dta*/+ ^mice.

**Figure 8:**
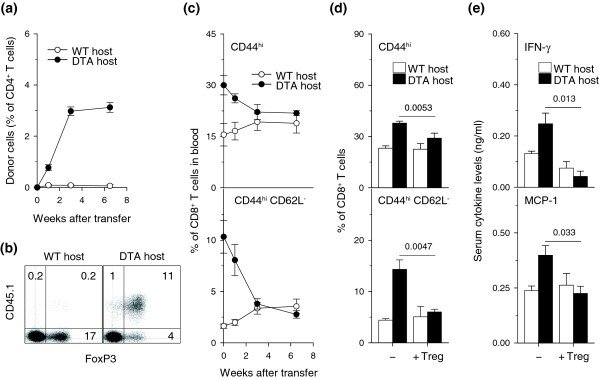
Restoration of immune homeostasis in *Tnfrsf4*^*Cre*/+^*R26*^*Dta*/+ ^mice by regulatory T cells. **(a) **Expansion of donor-type T cells in the blood of *Tnfrsf4*^*Cre*/+^*R26*^*Dta*/+ ^(DTA host) and control *Tnfrsf4*^*Cre*/+^*R26*^+/+ ^(WT host) recipients of purified regulatory CD45.1^+^CD25^+^CD4^+ ^T cells. **(b) **Expansion and retention of FoxP3 expression in donor CD45.1^+^CD4^+ ^T cells. Plots show gated CD4^+ ^T cells from lymphoid organs of recipient mice at the end of a 10-week observation period. **(c) **Percentage of CD44^hi^(top) and CD62L^-^CD44^hi ^(bottom) CD8^+ ^T cells in the blood of the same recipients of regulatory CD25^+^CD4^+ ^T cells. **(d) **Absolute numbers of CD44^hi ^(top) and CD62L^-^CD44^hi^(bottom) CD8^+ ^T cells in lymphoid organs of either DTA and WT mice that did not receive regulatory CD25^+^CD4^+ ^T cells (-) or DTA and WT mice 10 weeks after transfer of CD25^+^CD4^+ ^T cells (+ Treg). Values in (a-d) represent the mean (± SEM) of 7-10 mice per group pooled from three independent experiments. **(e) **Mean serum levels (± SEM) of IFN-γ and MCP-1 in the same mice. Similar results were obtained for IL-12p40, MIP-1α and IL-1β.

A deficit in Treg cells has been linked by some studies to immune activation and disease progression in HIV infection [29,30]. We therefore examined whether immune activation in *Tnfrsf4*^*Cre*/+ ^*R26*^*Dta*/+ ^mice resulted from Treg cell insufficiency. Wild-type regulatory CD4^+ ^T cells adoptively transferred into *Tnfrsf4*^*Cre*/+^*R26*^*Dta*/+ ^mice, but not into CD4^+ ^T cell-replete control mice, expanded efficiently, reaching numbers comparable to the number of regulatory CD4^+ ^T cells in wild-type mice, and maintained expression of FoxP3, the master regulator of Treg cell differentiation (Figure 8a, b), revealing a homeostatic deficit in the Treg cell pool of *Tnfrsf4*^*Cre*/+ ^*R26*^*Dta*/+ ^mice. The adoptively transferred regulatory CD4^+ ^T cells had a significant effect on the proportion of memory CD8^+ ^T cells, particularly those with an activated CD44^hi^CD62L^- ^phenotype, in *Tnfrsf4*^*Cre*/+^*R26*^*Dta*/+ ^mice; the proportion returned to wild-type levels over a period of 3 weeks (Figure 8c, d). Regulatory T cell reconstitution was also associated with reduction of pro-inflammatory cytokine serum levels in *Tnfrsf4*^*Cre*/+ ^*R26*^*Dta*/+ ^mice to levels comparable to those of control mice (Figure 8e). In addition to Treg cell insufficiency, *Tnfrsf4*^*Cre*/+ ^*R26*^*Dta*/+ ^mice were also characterized by impaired effector CD4^+ ^T cell function. To examine whether reduction of immune activation in *Tnfrsf4*^*Cre*/+^*R26*^*Dta*/+ ^mice upon transfer of wild-type CD4^+ ^T cells was specific to Treg cells, we performed similar adoptive transfer experiments using total wild-type CD4^+ ^T cells, consisting predominantly of non-regulatory conventional CD4^+ ^T cells, which gave rise to effector CD4^+ ^T cells. Although wild-type effector CD4^+ ^T cells efficiently reconstituted *Tnfrsf4*^*Cre*/+^*R26*^*Dta*/+ ^mice, but not control mice, they had no effect on the proportion of memory CD8^+ ^T cells or activated CD44^hi^CD62L^-^CD8^+ ^T cells (Additional data file 6). Reconstitution of *Tnfrsf4*^*Cre*/+ ^*R26*^*Dta*/+ ^mice with wild-type effector CD4^+ ^T cells also failed to rescue the reduced host Treg cell numbers in *Tnfrsf4*^*Cre*/+ ^*R26*^*Dta*/+ ^mice (Additional data file 7). Thus, immune activation in *Tnfrsf4*^*Cre*/+ ^*R26*^*Dta*/+ ^mice was due to a deficit in regulatory, but not effector, CD4^+ ^T cell function.

## Discussion

The complex and contrasting immune alterations in HIV infection are currently thought to have distinct etiologies [11,32]. Instead, our results suggest that the complexity of immune dysfunction in infection with HIV may simply reflect the functional heterogeneity of its targets. Conditional virus-free killing of activated CD4^+ ^T cells, which include both memory and regulatory subsets, was directly responsible for the development not only of immune deficiency, but also of activation.

As a result of the expression pattern of their receptors/co-receptors [13-15], replication of immunodeficiency viruses is largely restricted to the activated fraction of CD4^+ ^T cells. The finding that viruses such as HIV and FIV, which use different combinations of receptors/co-receptors to infect activated CD4^+ ^T cells, cause similar disease [13-15] indicates that specificity for target cells is more important for disease development than the cellular receptor conferring this specificity. Another important determinant of the rate of disease progression is the age at which HIV infection is acquired. Neonates generally develop symptomatic infection faster than adults, possibly because of the relative immaturity of the neonatal immune system and thus its inability to fully respond to the infection. Although a difference in an antiviral immune response would not influence the phenotype resulting from DTA-mediated CD4^+ ^T cell killing between neonate and adult mice, other factors may influence its severity. A limitation of the current approach is that CD4^+ ^T cell killing by CD134-driven DTA activation starts as soon as activated T cells are generated (within the first 3 days of birth in mice), which would thus correspond only to neonatal HIV infection. It would be important, once the tools become available, to compare the effect of DTA-mediated CD4^+ ^T cell killing in neonate and adult mice.

During the chronic phase of HIV or SIV infection only a small proportion of total CD4^+ ^T cells thought to be susceptible have been found to be infected at any one time [33]. In contrast, studies with SIV in rhesus macaques have revealed that up to 50% of all memory CD4^+ ^T cells are systemically killed during acute SIV infection [34,35]. Similarly, although approximately 50% of memory CD4^+ ^T cells were cumulatively marked by selection-neutral YFP expression in *Tnfrsf4*^*Cre*/+^*R26*^*Yfp*/+ ^mice, the potential for DTA-mediated death upon CD4^+ ^T cell activation in *Tnfrsf4*^*Cre*/+^*R26*^*Dta*/+ ^mice had surprisingly little effect on memory CD4^+ ^T cell survival and homeostasis. The reasons for this apparent 'resistance' to virus-mediated killing of susceptible CD4^+ ^T cell targets during the chronic phase of HIV infection are not known, but may be related to the naturally short lifespan of activated CD4^+ ^T cells, even when uninfected. The lifespan of HIV-infected CD4^+ ^T cells (the interval between virus entry and T cell death) has been estimated to be about 48 hours [36], which is very similar to the lifespan of activated CD4^+ ^T cells in *Tnfrsf4*^*Cre*/+ ^*R26*^*Dta*/+ ^mice (there is about a 48 hour interval between T cell activation and DTA-mediated death). It has been postulated that HIV replication is mostly restricted to relatively short-lived cellular targets [11], and it is therefore possible that the high natural turnover of activated CD4^+ ^T cells masks virus-induced death. Alternatively, the apparent 'resistance' of activated CD4^+ ^T cells during chronic HIV infection may represent selection for true HIV resistance in the CD4^+ ^T cell population [37].

Despite being the major target of virus replication, the proportion of CCR5^+^CD4^+ ^T cells paradoxically increases during less pathogenic HIV and SIV infection [11]. It may thus be unsurprising that despite efficient killing of memory CD4^+ ^T cells, their numbers in *Tnfrsf4*^*Cre*/+ ^*R26*^*Dta*/+ ^mice are preserved or even elevated under conditions of low thymic output. Although it will be important to establish why memory CD4^+ ^T cell replenishment eventually fails in more pathogenic HIV and SIV infection, our results also indicate that there is still CD4^+ ^T cell immunodeficiency even though numbers of memory CD4^+ ^T cells are not reduced at the population level. Studies in HIV infection have established that susceptibility to different infections is related to the degree of reduction in CD4^+ ^T cell counts in the blood [38]. These findings could suggest that protection against different infections requires a different number of CD4^+ ^T cells. Alternatively, susceptibility to different infections at different CD4^+ ^T cell counts could indicate a progressive decline in other arms of the adaptive immune system, especially CD8^+ ^T cells, a decline that correlates with the decline in CD4^+ ^T cells. The finding that *Tnfrsf4*^*Cre*/+^*R26*^*Dta*/+ ^mice show immunodeficiency in assays for CD4^+ ^T cell-mediated protection suggests that apart from the total number of memory CD4^+ ^T cells, the lifespan of individual clones and the clonal composition of the total memory pool are also crucial for immune competence.

In addition to immunodeficiency, conditional deletion of activated/memory CD4^+ ^T cells by CD134-driven DTA activation also leads to generalized immune activation, which shares many features with HIV infection-associated immune activation. Several distinct mechanisms have recently been proposed to underlie immune activation in HIV infection. A strong innate response to HIV components is thought to contribute to generalized immune activation and an attenuated innate response has been correlated with the non-pathogenic nature of SIV infection in sooty mangabeys [39]. Incomplete removal of apoptotic material as a result of accelerated T cell death in HIV infection has been proposed to induce self-reactive CD8^+ ^T cells [28]. HIV infection induces an early and extensive depletion of effector CD4^+ ^T cells at the intestinal mucosa, and it is thought that diminishing local immunity permits translocation of microbial products, which in turn causes generalized immune activation [32]. Lastly, the regulatory subset of CD4^+ ^T cells is targeted by HIV, SIV and FIV [40-43] and a relative deficit in this subset has been linked by certain studies to immune activation and disease progression [29,30,40,42,44,45].

Our results support a model in which generalized immune activation originates primarily from a relative insufficiency in Treg cells. The immune activation that develops in *Tnfrsf4*^*Cre*/+ ^*R26*^*Dta*/+ ^mice is diminished upon restoration of CD4^+ ^T cell homeostasis by wild-type CD4^+ ^T cells in bone marrow chimeras, demonstrating that immune activation in these mice results from dysregulated CD4^+ ^T cell homeostasis. The two subsets of CD4^+ ^T cells that are affected in *Tnfrsf4*^*Cre*/+ ^*R26*^*Dta*/+ ^mice include memory/effector and regulatory CD4^+ ^T cells, whereas naïve CD4^+ ^T cells are unaffected, suggesting that immune activation is due to insufficiency in either memory/effector or regulatory CD4^+ ^T cells, or both (general activated CD4^+ ^T cell lymphopenia). Treg cell numbers are reduced in *Tnfrsf4*^*Cre*/+ ^*R26*^*Dta*/+ ^mice, and the degree of relative Treg cell-specific lymphopenia in these mice is fully revealed by the expansion of adoptively transferred wild-type Treg cells. Furthermore, adoptive transfer of wild-type Treg cells reduces the immune activation seen in untreated *Tnfrsf4*^*Cre*/+^*R26*^*Dta*/+ ^mice. In contrast, adoptive transfer of total CD4^+ ^T cells, consisting largely of memory/effector CD4^+ ^T cells, does not appreciably reduce immune activation in *Tnfrsf4*^*Cre*/+^*R26*^*Dta*/+ ^mice. Lastly, immune activation in *Tnfrsf4*^*Cre*/+ ^*R26*^*Dta*/+ ^mice bears many similarities to the inflammatory disease that develops in mice with genetic deficiency in Treg cells [46]. Together, these findings indicate a causal link between Treg cell insufficiency and generalized immune activation in *Tnfrsf4*^*Cre*/+ ^*R26*^*Dta*/+ ^mice.

It is now clear that Treg cells are targeted by HIV, SIV and FIV [40-43]. However, their fate during infection remains controversial. The presence of Treg cell activity can be demonstrated during progression of HIV or SIV infection, and several studies have suggested that Treg cells are numerically increased and functionally activated during infection [29,47-50]. In contrast, other studies have indicated that Treg cells are lost during progression of HIV and SIV infection and shown a correlation between this loss and immune activation [29,30,40,42,44,45]. Given that Treg cell homeostasis relies mainly on growth factors, such as interleukin (IL)-2, produced by activated effector T cells, loss of effector CD4^+ ^T cells during pathogenic HIV or SIV infection would be expected to contribute to the loss of Treg cells, in addition to virus-mediated destruction. Loss of Treg cells in our model seems to be mainly due to intrinsic CD134-driven DTA activation, rather than loss of effector CD4^+ ^T cells. Firstly, expansion and maintenance of wild-type Treg cells adoptively transferred into *Tnfrsf4*^*Cre*/+ ^*R26*^*Dta*/+ ^mice is fully supported, indicating sufficient provision of Treg cell growth factors in these mice. Secondly, Treg cells of *Tnfrsf4*^*Cre*/+^*R26*^*Dta*/+ ^origin are severely reduced in numbers in bone marrow chimeras between *Tnfrsf4*^*Cre*/+ ^*R26*^*Dta*/+ ^and wild-type cells, despite the presence of normal numbers of wild-type effector CD4^+ ^T cells in these chimeric mice. Lastly, reconstitution of effector T cells, by adoptive transfer of wild-type CD4^+ ^T cells into *Tnfrsf4*^*Cre*/+ ^*R26*^*Dta*/+ ^mice, fails to restore the numbers of host Treg cells, suggesting that the defect in their homeostasis is intrinsic and not due to lack of effector CD4^+ ^T cells.

Thus, although a role for Treg cells in HIV disease progression is highlighted by all studies, it has remained unclear whether HIV infection is facilitated by excessive or insufficient Treg cell activity. Differences in methodology used to quantify Treg cells notwithstanding, whether Treg cell activity is increased or lost during progression of HIV infection will also depend on the behavior of cells that need to be regulated. Indeed, expansion and activation of Treg cells is not at odds with insufficient regulation if expansion and activation of effector CD4^+ ^and CD8^+ ^T cells is disproportionally higher. Furthermore, studies in natural hosts of SIV have suggested that preservation or functional redundancy of regulatory subsets could be responsible for the lack of immune activation and disease progression in non-pathogenic SIV infection [42,51].

The immune alterations that arise from conditional ablation of activated CD4^+ ^T cells in the system used here do not reproduce the entire spectrum of immune dysfunction that characterizes the various stages of HIV infection, indicating a multifactorial origin. Nevertheless, the enlargement of the lymph nodes, elevated serum levels of pro-inflammatory cytokines and chemokines, hyperplasia of the B cell compartment and increased T cell turnover and activation in *Tnfrsf4*^*Cre*/+ ^*R26*^*Dta*/+ ^mice lend further support to the idea that generalized immune activation may result from Treg cell insufficiency. However, it also raises the question of why immune activation in the setting of HIV infection and in *Tnfrsf4*^*Cre*/+ ^*R26*^*Dta*/+ ^mice is not associated with overt autoimmunity, as it is in Treg cell-deficient mice [46]. Perhaps disastrous autoimmunity develops during complete Treg cell deficiency, whereas HIV infection and *Tnfrsf4*^*Cre*/+ ^*R26*^*Dta*/+ ^mice show only partial Treg cell loss. Furthermore, most of the effector CD4^+ ^T cells that would otherwise mediate self-tissue damage are also targeted in HIV infection and in *Tnfrsf4*^*Cre*/+^*R26*^*Dta*/+ ^mice, and thus a substantial pathogenic component is removed.

Collectively, our results support a model for HIV pathogenesis in which immune deficiency and activation originate from virus-mediated killing of memory and regulatory CD4^+ ^T cells, respectively. According to the proposed model, generalized immune activation is a consequence, rather that the cause, of accelerated CD4^+ ^T cell turnover. Nevertheless, once instigated, immune activation will also contribute to the progressive loss of CD4^+ ^T cells by completing the cycle of cell activation and death. Defining the precise balance between CD4^+ ^T cell killing and immune activation and deficiency will be vital to our understanding of the pathogenesis of immune deficiency virus infection and to any effort to influence its outcome in favor of the host.

## Materials and methods

### Mice

Inbred C57BL/6 (B6) and CD45.1-congenic B6 mice (B6.SJL-*Ptprc*^*a*^*Pep3*^*b*^/BoyJ) were originally obtained from the Jackson Laboratory (Bar Harbor, USA) and were subsequently maintained at NIMR. B6-backcrossed Rag1-deficient mice [52] (B6.129S7-*Rag1*^*tm*1*Mom*^/J, called *Rag1*^-/-*here*)^, T cell receptor α (TCRα)-deficient mice [53] (B6.129-*Tcra*^*tm*1*Phi*^, called *Tcra*^-/-^), MHC II-deficient mice [54] (B6.129S2-*H2*^*dlAb*1-*Ea*^/J, called MHC II^-/-^) and MyD88-deficient mice [55] (B6.129-*Myd88*^*tm*1*Aki*^, called *Myd88*^-/-^) have been previously described and have also been maintained at NIMR. Mice with an activatable gene encoding YFP targeted into the ubiquitously expressed *Gt(ROSA)26Sor *(*R26*) locus have been described [27] and were backcrossed onto the B6 genetic background for at least ten generations.

Mice with an activatable gene encoding DTA targeted into the *R26 *locus were generated by gene targeting in embryonic stem cells. Briefly, the pUC-DTA vector containing a truncated gene encoding DTA (amino acids 3-193), in which the initial Met-Asp-Pro sequence is donated by the human metallothionein IIA, and which is followed by an SV40 carboxy-terminal Ser-Leu and small t intron, was kindly provided by Ian Maxwell, University of Colorado, Denver, USA. This fragment was subsequently inserted into the pBigT cassette, which also contained a loxP-flanked (floxed) neomycin-resistance gene (neo) and a triple poly-adenylation signal (tpA). The floxed neo-tpA-DTA fragment was subcloned into the RODA26PA vector, which was then linearized and electroporated into R1 embryonic stem cells. Targeted embryonic stem cell clones were injected into B6 blastocysts and germline transmitting *R26*^*Dta*/+ ^mice were backcrossed onto the B6 genetic background for at least six generations.

Mice with a targeted insertion of Cre recombinase into the *Tnfrsf4 *locus were generated by gene targeting in embryonic stem cells [26]. *Tnfrsf4*^*Cre *^mice were backcrossed onto the B6 genetic background for at least six generations. To obtain *Tnfrsf4*^*Cre*/+^*R26*^*Dta*/+ ^progeny, homozygous *Tnfrsf4*^*Cre*/*Cre *^mice were mated with heterozygous *R26*^*Dta*/+ ^mice. *Tnfrsf4*^*Cre*/+ ^*R26*^*Dta*/+ ^mice were born at expected Mendelian ratios and were viable with no clinical or histological signs of disease or pathology. A small proportion (<8%) of *Tnfrsf4*^*Cre*/+^*R26*^*Dta*/+ ^mice showed retarded development, which was evident as early as weaning. Histopathology analysis revealed exocrine pancreatic atrophy, consistent with the animals' small size, with absence of inflammatory infiltrates. All remaining organs, including endocrine pancreas, were normal. These mice were excluded from further analysis. In all experiments *Tnfrsf4*^*Cre*/+ ^*R26*^+/+ ^littermates were included as controls for *Tnfrsf4*^*Cre*/+^*R26*^*Dta*/+ ^mice to control for any potential effects of CD134-hemizygosity.

Mice with intestinal epithelial cell-specific deletion of *Ikbkg *(encoding IKKγ, also called NEMO) were obtained by crossing mice with a Cre-deletable *Ikbkg *allele (*Ikbkg*^*fl*^) with mice expressing Cre under the intestinal epithelial-specific villin promoter (*Vil-Cre*) and have been previously described [31]. *Ikbkg*^*fl*/*Y *^*Vil-Cre *and control *Ikbkg*^*fl*/*Y *^mice were bred and maintained at the Institute for Genetics, Cologne, Germany. Spleens and lymph nodes from *Ikbkg*^*fl*/*Y*^*Vil-Cre *and control *Ikbkg*^*fl*/*Y *^male mice were harvested at Cologne and shipped in Iscove's modified Dulbecco's medium (IMDM) to NIMR, where they were analyzed the following day. All animal experiments were conducted according to local government regulations and institutional guidelines.

### Infections

The A/PR/8/34 (PR8) strain of influenza A virus (IAV; kindly provided by Rose Gonsalves, Division of Virology, NIMR) was an allantoic fluid preparation from PR8-infected embryonated eggs. Non-anesthetized mice were infected with 250 hemagglutinin units of PR8 by instillation onto their nasal cavities. Serum titers of IAV-neutralizing antibodies were measured as previously described [56]. The Friend virus (FV) used in this study is a retroviral complex of a replication-competent B-tropic helper murine leukemia virus (F-MuLV-B) and a replication-defective polycythemia-inducing spleen focus-forming virus, referred to as FV. The FV stock (kindly provided by Kim Hasenkrug, Laboratory of Persistent Viral Diseases, Rocky Mountain Laboratories, NIAID, NIH, Hamilton, USA) was free of lactate dehydro-genase-elevating virus and was obtained as previously described [57].

FV was propagated *in vivo *and prepared as 10% w/v homogenate from the spleen of 12-day infected BALB/c mice. Mice received an inoculum of FV complex containing 1,000-2,000 spleen focus-forming units injected via the tail vein in 0.1 ml of phosphate-buffered saline. Cell-associated virus in infected mice was estimated by flow cytometric detection of infected cells using surface staining for the glycosylated product of the viral gag gene (glyco-Gag), using the matrix-specific monoclonal antibody 34 (mouse IgG2b), followed by an anti-mouse IgG2b-fluorescein isothiocyanate (FITC) secondary reagent (BD Biosciences, San Jose, USA). Serum titers of FV-neutralizing antibodies were measured as previously described [57].

*Pneumocystis murina *was obtained from ATCC/LGC Promochem (stock PRA-111) and administered to non-anesthetized mice by instillation onto their nasal cavities. *P. murina *was detected in formalin-fixed lung tissue by Gomori's silver stain of lung sections by IZVG Pathology (Leeds, UK) and in fresh lung tissue by PCR specific for the *P. murina *mitochondrial large-subunit rRNA gene [58].

### Flow cytometry

Cells were stained with directly conjugated antibodies to surface markers, obtained from eBiosciences (San Diego, USA), CALTAG/Invitrogen (Carlsbad, USA) or BD Biosciences. B cells and macrophages were identified as B220^+ ^and CD11b^+^F4/80^+ ^cells, respectively. Naïve, memory and regulatory T cells were identified as CD44^lo^CD25^-^, CD44^hi^CD25^- ^and CD25^+ ^cells, respectively. Four- and eight-color cytometry were performed on FACSCalibur (BD Biosciences) and CyAn (Dako, Fort Collins, USA) flow cytometers, respectively, and analyzed with FlowJo v8.7 (Tree Star Inc., Ashland, USA) or Summit v4.3 (Dako) analysis software, respectively. For detection of cytokine synthesis, cells were stained for surface markers and stimulated for 4 h with phorbol 12,13-dibutyrate and ionomycin (both at 500 ng/ml), in the presence of monensin (1 μg/ml). Cells were then fixed and permeabilized using buffers from eBiosciences, before intracellular staining with tumor necrosis factor (TNF)-α- and interferon (IFN)-γ-specific antibodies (eBiosciences). FoxP3 was detected by intranuclear staining using a FoxP3-staining kit (eBiosciences) according to the manufacturer's instructions. Apoptotic cells were stained using an Annexin V staining kit (BD Biosciences) according to the manufacturer's instructions. Cellular turnover was assessed by BrdU incorporation during a 6-day administration into the drinking water of mice, and in addition 3 days after cessation of BrdU administration. BrdU incorporation was measured using a BrdU staining kit (BD Biosciences) according to the manufacturer's instructions. Ki67-expressing cells were identified using an anti-human Ki67 or matched isotype control (clones B56 and MOPC-2, respectively, BD Biosciences).

### Cell isolation, purification, labeling and transfer

Single cell suspensions were prepared from thymus, spleen or lymph nodes of donor mice by mechanical disruption. Spleen suspensions were treated with ammonium chloride for erythrocyte lysis. Lymph node cellularity was calculated as the sum of the cellular contents of inguinal, axillary, brachial, mesenteric and superficial cervical lymph nodes. Total cell numbers of the various lymphoid and myeloid subsets were the sum of the splenic and lymph node content.

Bone marrow cell suspensions were prepared by flushing the bone cavities of femurs and tibiae from donor mice with IMDM. Target cells were enriched in lymph node and spleen suspensions using immunomagnetic positive selection (EasySep beads, StemCell Technologies, Vancouver, Canada) according to the manufacturer's instructions. Enriched cell suspensions were stained with antibodies to surface markers and then further purified by cell sorting, performed on MoFlo cell sorters (Dako). Typical cell purity following cell sorting was higher than 98%. In some experiments, purified cells were further labeled with CFSE (Molecular Probes/Invitrogen, Carlsbad, USA). Purified cells (1 × 10^6 ^per recipient for unlabeled cells or 5 × 10^6 ^per recipient for CFSE-labeled cells) were injected into recipient mice through the tail vein in 0.1 ml of air-buffered IMDM.

### Bone marrow chimeras

CD45.2^+ ^*Tnfrsf4*^*Cre*/+ ^*R26*^*Dta*/+ ^(DTA) and CD45.1^+ ^C57BL/6 (B6) wild-type bone marrow cells were injected separately or mixed together (mixed bone marrow chimeras) into non-irradiated *Rag1*^-/- ^recipients expressing the allotypic marker CD45.2. Each recipient received one mouse-equivalent of bone marrow cells. Mice were bled periodically for assessment of reconstitution and lymphoid organs were analyzed 12 weeks after bone marrow transfer. In separate experiments, CD45.1^+ ^B6 bone marrow cells were injected into non-irradiated CD45.2^+ ^*Rag1*^-/- ^recipients and reconstitution of lymphoid, myeloid and erythroid lineages by donor-type cells was assessed. This analysis revealed that in non-irradiated *Rag1*^-/- ^recipients only the lymphoid lineage (T and B cells) is reconstituted by donor-type cells, whereas all other lineages were still host-derived.

### *In vitro *T cell activation

Single cell suspensions were prepared from the spleen or lymph nodes of donor mice and 0.5 × 10^6 ^purified T cells per well were stimulated in 96-well plates with CD3/CD28-coated beads (Mouse CD3/CD28 T Cell Expander, Dynal/Invitrogen, Carlsbad, USA) at 1:1 ratio for the indicated length of time.

### Serum cytokines and LBP

Levels of serum cytokines were assessed by multiplex cytokine bead arrays (20-plex Panel, BioSource/Invitrogen, Carlsbad, USA; or 23-plex Panel, Bio-Rad, Hercules, USA) using the Luminex 100 System (Luminex, Austin, USA). Levels of serum LBP were determined by ELISA (HyCult Biotech, Uden, The Netherlands) according to manufacturer's instructions.

### Statistical analysis

Parametric comparisons were made by Student's *t*-test performed using SigmaPlot v10 software (Systat Software Inc., San Jose USA). Fisher's exact test was used specifically for the non-parametric comparison of *P. murina*-infected and non-infected mice.

## Supplementary Material

Additional file 1 Strategy for targeting of an activatable DTA-encoding gene into the *R26 *locus. Click here for file

Additional file 2 DTA-mediated deletion of memory and regulatory CD4^+ ^T cells in mixed bone marrow chimeras. Click here for file

Additional file 3 CD4^+ ^T cell number dependency of influenza A virus (IAV)-neutralizing antibody induction. Click here for file

Additional file 4 CD8^+ ^T cell number and phenotype in MHC II-deficient mice. Click here for file

Additional file 5 Effect of microbial translocation on lymphocyte turnover in *Ikbkg*^*fl*/*Y *^*Vil-Cre *mice. Click here for file

Additional file 6 Effect of CD4^+ ^T cell reconstitution on CD8^+ ^T cell activation in *Tnfrsf4*^*Cre*/+ ^*R26*^*Dta*/+ ^mice. Click here for file

Additional file 7 Effect of CD4^+ ^T cell reconstitution on Treg cell number in *Tnfrsf4*^*Cre*/+ ^*R26*^*Dta*/+ ^mice. Click here for file
