# Supplementary material for: Generalized immune activation as a direct result of activated CD4+ T cell killing
Source: J Biol. 2009 Nov 27;8(10):93. doi: 10.1186/jbiol194 (PMC2790834; doi:10.1186/jbiol194)
Supplement: Additional file 1 — Strategy for targeting of an activatable DTA-encoding gene into the R26 locus. [file jbiol194-S1.pdf]

Additional data file 1

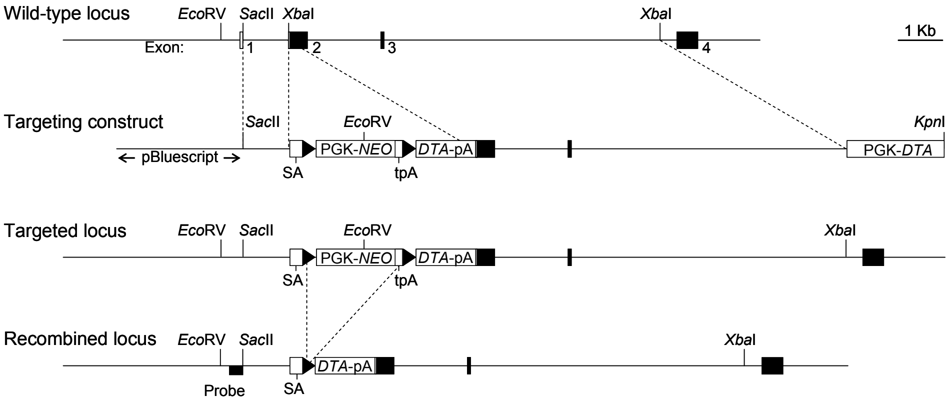

Additional figure 1. Strategy for targeting of an activatable DTA-encoding gene into the *R26* locus. Schematic representation of the wild-type *R26* locus, targeting construct and targeted locus, before and after Cre-mediated recombination. Coding R26 exons are indicated by filled boxes. Filled triangles represent *loxP* sites. Relevant restriction sites are indicated. SA, splice acceptor; pA polyadenylation site; tpA, triple pA.
