# Supplementary material for: Generalized immune activation as a direct result of activated CD4+ T cell killing
Source: J Biol. 2009 Nov 27;8(10):93. doi: 10.1186/jbiol194 (PMC2790834; doi:10.1186/jbiol194)
Supplement: Additional file 2 — DTA-mediated deletion of memory and regulatory CD4+ T cells in mixed bone marrow chimeras. [file jbiol194-S2.pdf]

Additional data file 2

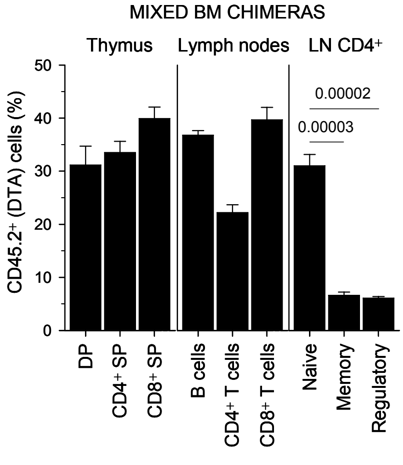

Additional figure 2. DTA-mediated deletion of memory and regulatory $CD4^+$ T cells in mixed bone marrow chimeras. $CD45.2^+$ *Tnfrsf4*$^{Cre/+}$ *R26*$^{Dta/+}$ (DTA) and $CD45.1^+$ C57BL/6 (B6) bone marrow (BM) cells were injected separately or mixed together (mixed BM chimeras) into non-irradiated *Rag1*$^{-/-}$ recipients and lymphoid organs were analyzed 12 weeks later. Mean (±SEM) percentage of DTA-origin ($CD45.2^+$) cells in thymocyte and peripheral lymphocyte subsets in mixed BM chimeras is shown. Values are representative of 4-8 mice analyzed in 2 independent experiments.
