# Supplementary material for: Generalized immune activation as a direct result of activated CD4+ T cell killing
Source: J Biol. 2009 Nov 27;8(10):93. doi: 10.1186/jbiol194 (PMC2790834; doi:10.1186/jbiol194)
Supplement: Additional file 3 — CD4+ T cell number dependency of influenza A virus (IAV)-neutralizing antibody induction. [file jbiol194-S3.pdf]

Additional data file 3

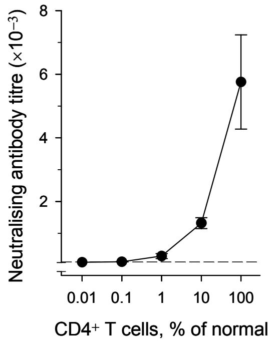

Additional figure 3. CD4$^+$ T cell number dependency of Influenza A virus (IAV)-neutralizing antibody induction. Titers of IAV-neutralizing antibodies in the serum of T cell-deficient *Tcra*$^{-/-}$ mice, which received titrated numbers of CD4$^+$ T cells from wild-type B6 donor mice are shown. Sera were tested 18 days post IAV infection. The X axis depicts the number of adoptively transferred CD4$^+$ T cells, on the day of the transfer, expressed as a percentage of the total number of CD4$^+$ T cells in wild-type B6 mice (100%). Symbols represent the mean (±SEM) of 3-4 mice.
