# Supplementary material for: Generalized immune activation as a direct result of activated CD4+ T cell killing
Source: J Biol. 2009 Nov 27;8(10):93. doi: 10.1186/jbiol194 (PMC2790834; doi:10.1186/jbiol194)
Supplement: Additional file 4 — CD8+ T cell number and phenotype in MHC II-deficient mice. [file jbiol194-S4.pdf]

Additional data file 4

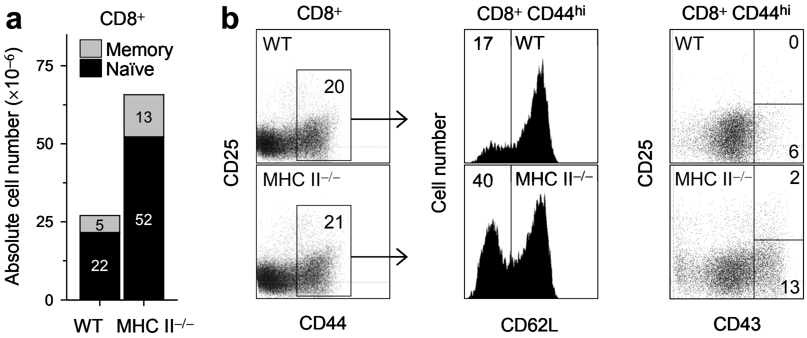

Additional figure 4. $CD8^+$ T cell number and phenotype in MHC II-deficient mice. (**a**) Absolute number (mean, *n*=4-6) of naïve and memory $CD8^+$ T cells in MHC II-deficient (MHC $II^{-/-}$) and wild-type control (WT) mice. (**b**) CD62L, CD43 and CD25 expression in gated memory $CD44^{hi}CD8^+$ T cells from the same mice.
