# Supplementary material for: Generalized immune activation as a direct result of activated CD4+ T cell killing
Source: J Biol. 2009 Nov 27;8(10):93. doi: 10.1186/jbiol194 (PMC2790834; doi:10.1186/jbiol194)
Supplement: Additional file 5 — Effect of microbial translocation on lymphocyte turnover in Ikbkgfl/Y Vil-Cre mice. [file jbiol194-S5.pdf]

Additional data file 5

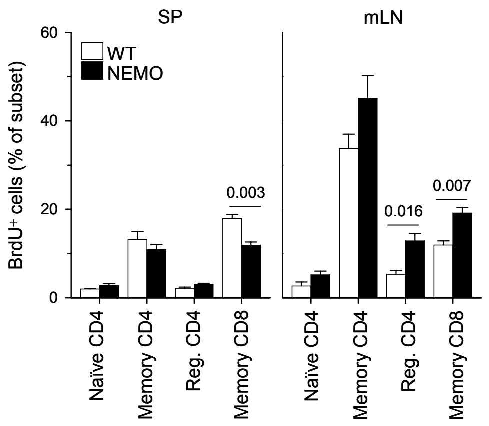

Additional figure 5. Effect of microbial translocation on lymphocyte turnover. BrdU incorporation during a 6-day administration period in naïve, memory and regulatory (Reg.) $CD4^+$ T cells and memory $CD8^+$ T cells from the spleens (SP, *left*) or mesenteric lymph nodes (mLN, *right*) of *Ikbkg*$^{fl/Y}$ *Vil-Cre* (NEMO) and littermate control *Ikbkg*$^{fl/Y}$ (WT) mice. Values are the mean (±SEM) of 3-4 mice per group.
