# Supplementary material for: Generalized immune activation as a direct result of activated CD4+ T cell killing
Source: J Biol. 2009 Nov 27;8(10):93. doi: 10.1186/jbiol194 (PMC2790834; doi:10.1186/jbiol194)
Supplement: Additional file 6 — Effect of CD4+ T cell reconstitution on CD8+ T cell activation in Tnfrsf4Cre/+ R26Dta/+ mice. [file jbiol194-S6.pdf]

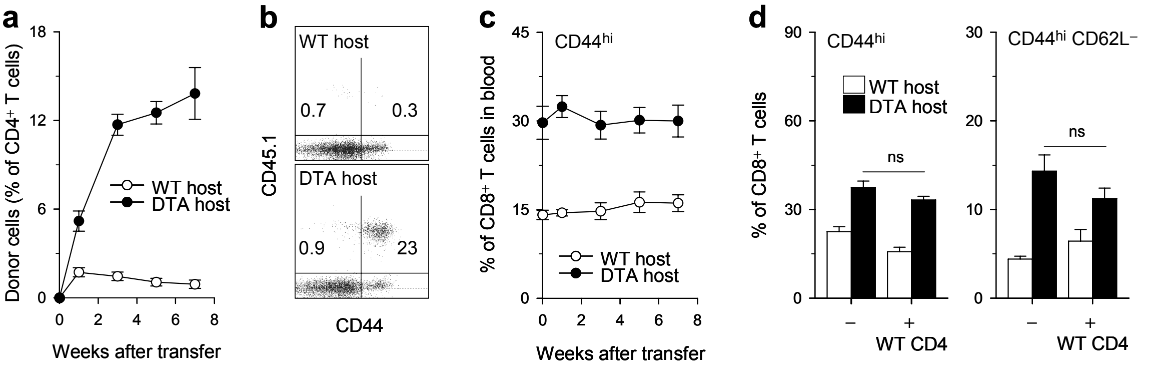

Additional figure 6. Effect of $CD4^+$ T cell reconstitution on $CD8^+$ T cell activation in $Tnfrsf4^{Cre/+}$ $R26^{Dta/+}$ mice. (**a**) Expansion of donor-type T cells in the blood of $Tnfrsf4^{Cre/+}$ $R26^{Dta/+}$ (DTA host) and control $Tnfrsf4^{Cre/+}$ $R26^{+/+}$ (WT host) recipients of purified wild-type $CD45.1^+CD4^+$ T cells. (**b**) Expansion of donor $CD45.1^+CD4^+$ T cells. Plots show gated $CD4^+$ T cells from lymphoid organs of recipient mice at the end of a 7-week observation period. (**c**) Percentage of memory cells ($CD44^{hi}$) in $CD8^+$ T cells in the blood of the same recipients of wild-type $CD45.1^+CD4^+$ T cells. (**d**) Absolute numbers of $CD44^{hi}$ (*left*) and $CD62L^-CD44^{hi}$ (*right*) $CD8^+$ T cells in lymphoid organs of DTA and WT mice, which did not receive $CD4^+$ T cells (-) or DTA and WT mice 7 weeks after transfer of wild-type $CD45.1^+CD4^+$ T cells (+ WT CD4). Values in (a) to (d) represent the mean (±SEM) of 5-6 mice per group pooled from 2 independent experiments.
