# Supplementary material for: Generalized immune activation as a direct result of activated CD4+ T cell killing
Source: J Biol. 2009 Nov 27;8(10):93. doi: 10.1186/jbiol194 (PMC2790834; doi:10.1186/jbiol194)
Supplement: Additional file 7 — Effect of CD4+ T cell reconstitution on Treg cell number in Tnfrsf4Cre/+ R26Dta/+ mice. [file jbiol194-S7.pdf]

Additional data file 7

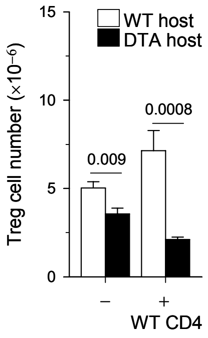

Additional figure 7. Effect of $CD4^+$ T cell reconstitution on Treg cell number in *Tnfrsf4*$^{Cre/+}$ *R26*$^{Dta/+}$ mice. Absolute numbers of Treg cells ($CD25^+CD4^+$) in lymphoid organs of DTA and WT mice, which did not receive $CD4^+$ T cells (-) or DTA and WT mice 7 weeks after transfer of wild-type $CD45.1^+CD4^+$ T cells (+ WT CD4). Values represent the mean (±SEM) of 5-6 mice per group pooled from 2 independent adoptive transfer experiments.
